# What Is Cincho-Quinine?

**Published:** 1874-02

**Authors:** James B. Baird

**Affiliations:** Atlanta


### WHAT IS CINCHO QUININE?

BY JAMES B. BAIRD, M.D.

THE ATLANTA MEDICAL AND SURGICAL JOURNAL, for October, contains an article, republished from the June, 1869, number, giving an unqualified indorsement to "Cincho Quinine," and claiming for it not only equally valuable therapeutic properties, but even declaring it superior in many, if not in all, respects to the sulphate of quinine.

The article in question unhesitatingly affirms that it contains quinia cinchonia, quinidia, cinchonidia, and other alkaloidal principles, which have not been distinctly isolated. That it exerts the full therapeutic influence of quinine in the same doses, does not oppress the stomach or create nausea. It does not produce cerebral distress, or so much "constitutional disturbance." It is nearly tasteless. It is not so expensive as quinine, and that "it meets indications not met by that salt."

As there are two sides to every question, the following extract from the October number of the *American Journal of Pharmacy,* presents the other side of the "Cincho-Quinine" question.

"What is chincho-quinine?" Under this caption an article has been published lately in many medical journals, laudatory of a preparation which has been chemically examined by W. T. Wenzell, of San Fransisco, whose results were published in the April number of 1870, of the "*Pacific Medical and Surgical Journal.*" For the benefit of our cotemporaries, we again copy here the concluding paragraghs of Mr. Wenzell's paper, which will furnish an answer to the above, or rather to the query, "What was cincho-quinine in March, 1870?"

"Cincho-quinine," although having the advantage of being nearly tasteless, does not contain quinia, quinidia and cinchonidia, and therefore does not represent the whole of the active principles of the bark. It cannot exert the full effects of sulphate of quinia in the same dose, inasmuch as the stated dose of "cincho-quinine" is from five to thirty grains.

Although "cincho-quinine" appears to cost less than sulphate of quinia, it does not follow that commercial cinchonia, sold at four times its value, is a desirable substitute for quinine in an economical point of view—and, lastly, one very important principle should, by no means, be lost sight of, namely, that a physician should always know what he is prescribing, and therefore the substitution of a remedy of less efficiency and uncertain medicinal value is unwarrantable and often hazardous."

Where testimony is so positive and so conflicting, the question at issue must remain for the present, at least, *sub judice.*

Atlanta, Nov. 18, 1873.

